# Excitonic Complexes in Two-Dimensional Transition Metal Dichalcogenides

**DOI:** 10.1038/s41467-023-44119-9

**Published:** 2023-12-12

**Authors:** Xiaotong Chen, Zhen Lian, Yuze Meng, Lei Ma, Su-Fei Shi

**Affiliations:** 1 Department of Chemical and Biological Engineering, Rensselaer Polytechnic Institute, Troy, NY 12180 USA; 2 Department of Physics, Carnegie Mellon University, Pittsburgh, PA 15213 USA

**Keywords:** Two-dimensional materials, Condensed-matter physics, Optical spectroscopy

## Abstract

The enhanced Coulomb interaction in two dimensions leads to not only tightly bound excitons but also many-particle excitonic complexes: excitons interacting with other quasiparticles, which results in improved and even new exciton properties with better controls. Here, we summarize studies of excitonic complexes in monolayer transition metal dichalcogenides and their moiré heterojunctions, envisioning how to utilize them for exploring quantum many-body physics.

Unlike their bulk counterparts, monolayer transition metal dichalcogenides (TMDCs) are direct bandgap semiconductors with superior optical properties, such as prominent photoluminescence (PL). The reduced screening in two-dimensional (2D) materials enhances Coulomb interaction and leads to tightly bound exciton with the binding energy on the order of 100 meV, robust enough to survive thermal fluctuations at room temperature^1^. The significant spin-orbit coupling (SOC) leads to a large splitting in the valence band of monolayer TMDC, ~150-500 meV. The strong SOC, combined with inversion symmetry breaking and three-fold rotation symmetry, results in a robust quantum degree of freedom, valley-spin, promising for the applications of valleytronics and quantum computing^1,2^.

The strong Coulomb interaction also allows the excitons to bind/interact with other quasiparticles such as exciton, electrons (holes), and phonons, giving rise to many-particle excitonic complexes such as trions, biexcitons, exciton-trion complexes, and dark exciton phonon replica. The unique interactions leading to the excitonic complexes can be utilized for engineering new excitonic states, ranging from valley-polarized excitonic complexes with an increased lifetime in monolayer TMDC to correlated excitons in TMDC moiré superlattices. The exciting opportunities ushered in by the excitonic complexes, therefore, call for the summary and discussion of the recent progress.

## Excitonic complexes in monolayer TMDCs

We use monolayer WSe_2_ as an archetype for the discussion because the effect of the excitonic complexes in it is enhanced due to the presence of spin-forbidden dark excitons, although the concept applies to TMDCs in general. There is a sizable SOC-induced splitting of conduction bands in monolayer TMDCs, about 3-35 meV, which is strikingly different for Mo-based and W-based TMDCs. In WSe_2_ and WS_2_, the conduction band minimum (CBM) possesses the opposite spin as the valence band maximum (VBM) of specific spin^1,2^. As a result, the ground state of the exciton is a long-lived spin-forbidden dark exciton with a high density even under moderate optical excitation power density, which increases the possibility of forming excitonic complexes^3^.

**Fig. 1:**
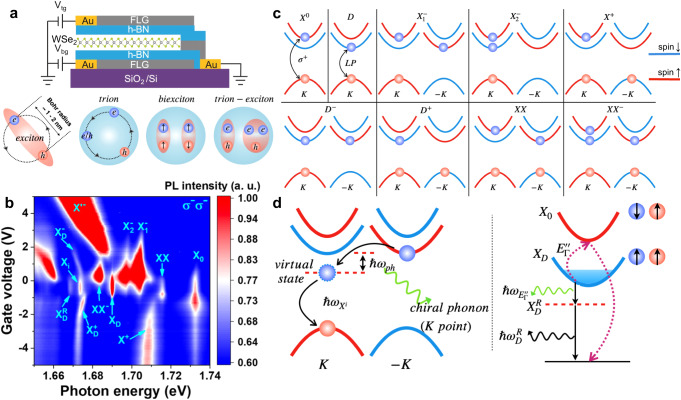
Excitonic complexes in monolayer TMDCs. **a** Schematics of a dual-gated BN encapsulated monolayer WSe_2_ device and excitonic complexes. **b** Doping-dependent Photoluminescence (PL) spectra of monolayer WSe_2_ at 4.2 K. Reproduced with permission from Ref. ^12^. Copyright 2019 American Chemical Society **c** Band configurations of excitonic complexes. **d** Schematics of the phonon replicas of intervalley excitons (left) and spin-forbidden dark excitons (right) in monolayer WSe_2_, along with phonon scattering process involving chiral phonons at K point and Γ point.

A typical monolayer WSe_2_ device with a doping tunability is schematically illustrated in Fig. 1a. Shown in Fig. 1b are doping-dependent photoluminescence (PL) spectra of monolayer WSe_2_ measured at 4.2 K, with each resonance representing an excitonic complex. The constitutions of various exciton complexes are also schematically represented in Fig. 1c. The PL peak X_0_ near 1.732 eV is the bright exciton, with the electron from the conduction band that has the same spin as the VBM, which is short-lived with a lifetime of about 1-10 ps due to its excited state nature^1,3^. X^+^ is the positive trion, an exciton bound to a free hole, with a binding energy of ~20 meV^1–3^. Both ${X}_{1}^{-}$ and ${X}_{2}^{-}$ are negative trions, an exciton bound to a free electron^2,3^. Due to the unique band structure of WSe_2_, the free electron can be at the CBM of the K’ or K valley, with the binding energy of 29 and 35 meV, respectively. The splitting of the ${X}_{1}^{-}$ and ${X}_{2}^{-}$ is due to the difference in the exchange interaction, which was calculated to be about 6 meV, in excellent agreement with the experimental observation^3^. It is worth noting that the trion is a rather simplified picture at relatively low doping. More rigorously, the bright exciton in monolayer TMDC will interact with the whole Fermi sea at the same or opposite valley, giving rise to repulsive and attractive exciton polaron, which are obviously in the reflectance and especially helicity-resolved reflectance spectra (not shown here)^4^. Exciton-polaron is a fascinating example of the many-body physics in TMDCs and has played an important role in identifying strongly correlated states recently^5^.

The PL peak labeled as XX is the biexciton resonance, and it corresponds to two excitons bound together due to the enhanced Coulomb interaction^1^, with a binding energy of ~20 meV^3^. The smaller binding energy than the trion is consistent with the expectation that the monopole-dipole would have stronger Coulomb attraction. Different from the linear power dependence of PL from excitons or trions, biexciton PL is supposed to exhibit quadratic power dependence. However, a super-linear power dependence with the power exponent between 1 and 2 is often experimentally observed due to the quasi-equilibrium nature of the excitons. The power exponent approaches 2 in high-quality samples. A Landé g-factor close to 4 for the XX peak was extracted from magneto-PL measurement. In addition, the higher energy branch from its Zeeman splitting carries more spectral weight than the lower energy one. From these observations, several research groups have concluded that a biexciton is composed of a bright exciton and a spin-forbidden dark exciton from the opposite valley, as shown in Fig. 1c, which explains why the biexciton is pronounced even at a moderate excitation power density^3,6–9^.

The XX^-^ is a biexciton bound to a free electron, which exhibits a similar super-linear power dependence as XX and emerges when the monolayer WSe_2_ is electron-doped. It can also be viewed as the combination of one exciton bound to a trion, hence an exciton-trion complex^3,6–9^.

The X_D_ is the spin-forbidden dark exciton, which is expected to have an out-of-plane dipole moment and emit photons that propagate in the plane. This unusual radiation pattern can be detected by collecting the PL emission from the edge of the sample or using an objective with a large numerical aperture (NA)^3^. Due to the reduced recombination rate of this dark state, the spin-forbidden dark exciton possesses a lifetime significantly longer than that of a bright exciton, which is around 250 ps according to time-resolved photoluminescence measurements^3^.

The ${X}_{D}^{+}$ and ${X}_{D}^{-}$ are the dark exciton trions, a spin-forbidden dark exciton bound to a free hole or electron. Their radiation pattern is shown to be the same as the dark exciton X_D_ (out-of-plane) via the back focal plane imaging^3^. Remarkably, the dark trions were found to possess a long valley lifetime greater than 3.5 ns, a result of the suppressed intervalley scattering due to the spin-configuration of the dark trions^10^.

The PL peak X^-^ was initially attributed to a plasmon mode but was recently assigned to six-particle states (“hexcitons”)^11^.

Apart from the spin-forbidden dark exciton, the intervalley exciton is another “dark” exciton, as the momentum mismatch of the electron and hole forbids the direct recombination of the electron-hole pair. The long lifetime of the intervalley and the spin-forbidden dark excitons (~250 ps), allow the photon replica of them that are also long-lived, ${X}_{i}$ (~200 ps) and ${X}_{D}^{R}$ (~230 ps)^3^. In both cases, the radiative recombination of dark excitons can be assisted by emitting a chiral phonon with a certain momentum (LO phonon at K point and E” phonon at Γ point, respectively), which provides a fixed pseudoangular momentum (PAM) to the optical transition, leading to the restoration of valley polarization of the dark excitons^3,12^. The chiral phonon scattering process is schematically illustrated in Fig. 1d. More phonon replica modes have also been found in even lower energy than what is shown in Fig. 1b, which is not shown here but can be found in Ref. ^13^ and ^14^.

Excitonic complexes could lead to excitons with improved properties and better controls. For example, excitonic complexes could lead to long-lived valley-polarized quasiparticles that are important for quantum information processing. Bright excitons in TMDCs are valley polarized but short-lived. In contrast, dark excitons could have longer lifetimes but lose valley polarization. Coupling dark excitons with chiral phonons could give rise to dark phonon replica modes that are valley-polarized and have lifetimes more than one order of magnitude longer (~200 ps) than bright excitons (~10 ps). The interaction between chiral phonons and excitons can be further exploited to manipulate the valley degree of freedom. With the improved understanding of interactions leading to the formation of excitonic complexes, we expect to see more exciting work in the near future on how to utilize these interactions to better control valley-spin, enhance nonlinearity, improve quantum efficiency, and even realize new excitonic physics.

## Excitonic complexes in TMDC moiré heterojunctions

**Fig. 2:**
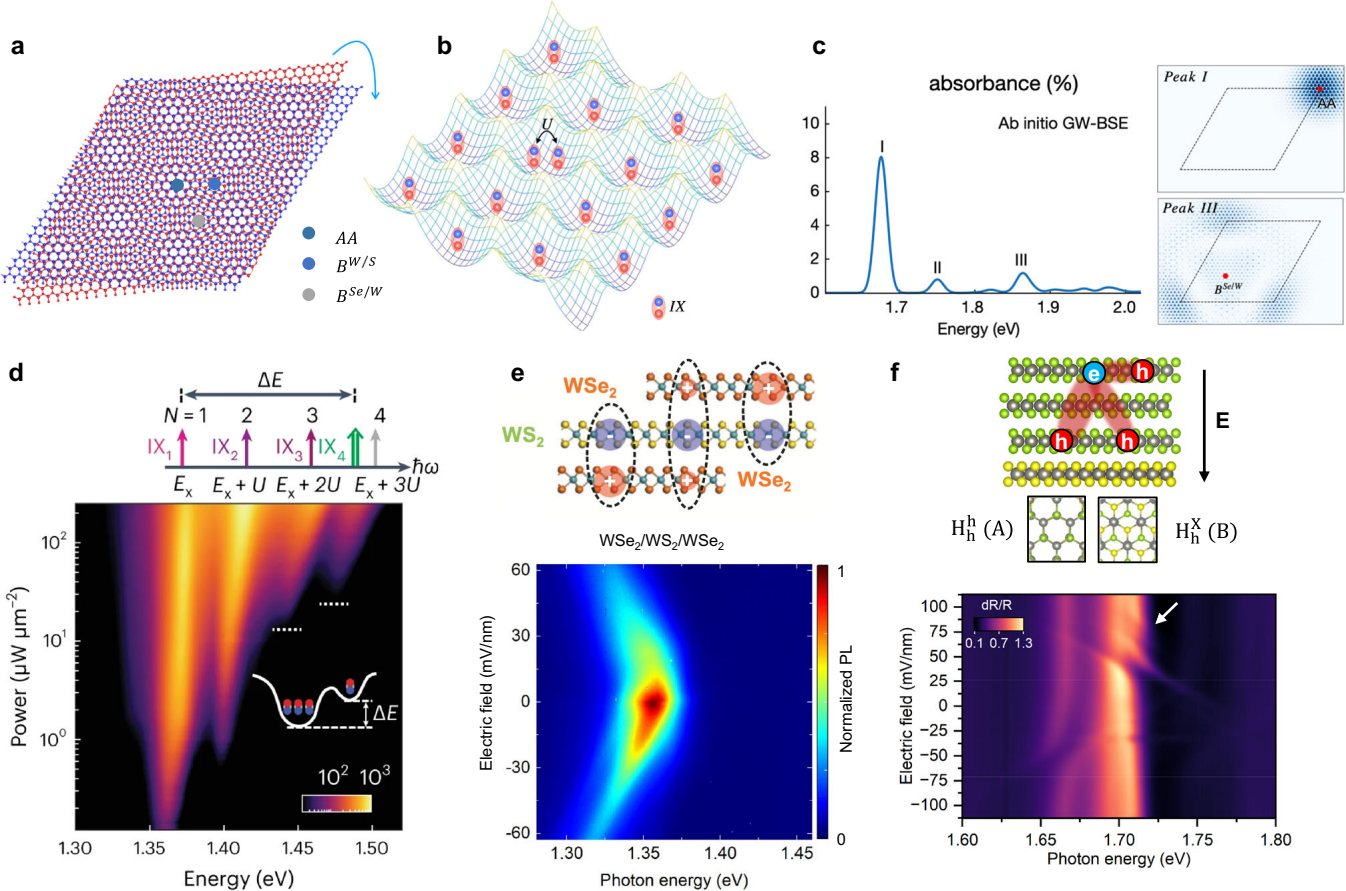
Excitonic complexes in TMDC moiré heterojunctions. **a** Schematic of the moiré heterojunction made of monolayer WSe_2_ and WS_2_, highlighting three high symmetry points. **b** Schematic of locally trapped excitons in a moiré superlattice. U denotes the onsite repulsion between excitons. **c** Calculated absorption spectrum (left) and wavefunctions (right) of the intralayer moiré excitons in the angle-aligned WSe_2_/WS_2_ moiré superlattices. Reproduced with permission from ref. ^19^. Copyright 2022 Springer Nature **d** Large Hubbard model U in WSe_2_/WS_2_ moiré superlattices. Reproduced with permission from ref. ^24^. Copyright 2023 Springer Nature. **e** Top: schematics of quadrupolar in WSe_2_/WS_2_/WSe_2_ hetero-trilayers and dipolar excitons in WSe_2_/WS_2_ heterobilayers. Bottom: PL spectra as a function of the out-of-plane electric field from a WSe_2_/WS_2_/WSe_2_ hetero-trilayer at 10 K. Reproduced from ref. ^29^ under a Creative Common license (http://creativecommons.org/licenses/by/4.0/). **f** Top: schematic of the three-level hybridized excitons in trilayer WSe_2_/monolayer WS_2_ moiré superlattices. Bottom: electric field dependence of the reflectance spectra at 10 K, the white arrow indicates the position where the hybridized exciton is observed. Reproduced from ref. ^35^ under a Creative Common license (http://creativecommons.org/licenses/by/4.0/).

Stacking two individual TMDC monolayers into heterobilayers, schematically shown in Fig. 2a, enables the control of exciton properties via band alignment engineering. TMDC heterobilayers with a type-II band alignment are known to host long-lived interlayer excitons, which consist of an electron in one layer and a hole in the other. Owing to its permanent electric dipole moment resulting from the electron-hole separation, the emission energy and the recombination dynamics of interlayer excitons are highly tunable by out-of-plane electric fields^15^. Theoretical calculations have shown that interlayer excitons exhibit distinct optical selection rules determined by spin, valley, and local stacking registry configurations^16,17^. As a result of these selection rules, the optical transition of spin-triplet interlayer excitons, whose recombination results from optical transitions between bands with opposite spins, could be brightened at certain lattice sites^5,15^.

Recent studies have shown that the presence of moiré superlattice in TMDC heterobilayers could significantly modulate the properties of excitons^5,18^. A moiré superlattice is formed by stacking two TMDC monolayers with a lattice mismatch or at a twist angle, with a periodic moiré potential significantly modifying the wavefunctions of carriers or excitons in the moiré lattice. A R-stacked WS_2_/WSe_2_ moiré superlattice is represented in Fig. 2a as an example where three high symmetry configurations (AA, B^W/S^, and B^Se/W^) emerge as the local potential extrema of the moiré potential. Due to the periodicity of the moiré potential landscape (Fig. 2b) in WS_2_/WSe_2_ heterobilayers, three moiré pattern-modulated intralayer exciton states located at these high symmetry points have been observed in absorption measurements^18^. Recent large-scale first-principles GW and Bethe–Salpeter calculation (Fig. 2c) suggests that the main peak I is located at AA point and peak III is a charge-transfer exciton with the hole located at B^Se/W^ and the electron at AA^19^.

In TMDC moiré heterobilayers, interlayer excitons are also expected to be trapped at the high symmetry points of the moiré lattice^5,18^. The attractive interaction between free carriers and moiré interlayer excitons has been reported to give rise to moiré trions^20,21^. The correlated electrons, such as those at the Mott insulator state, however, can have repulsive interaction with interlayer excitons and lead to a significant blueshift of interlayer exciton PL^22^. Further, the strong onsite dipolar repulsion between interlayer excitons also gives rise to a correlated insulator of excitons at an exciton density corresponding to one exciton per moiré unit cell (Fig. 2d), which can be described by the bosonic Hubbard model and is evident by the observation of an excitonic incompressible state and a large Hubbard model U around 30-40 meV in WSe_2_/WS_2_ moiré superlattice^22–24^.

It is worth noting that the inter-moiré superlattice electron repulsion (V) is also strong, which leads to the extended Hubbard model and generalized Wigner crystal states at fractional fillings (multiple moiré superlattice sharing one electron or hole). The doping-dependent PL spectra of interlayer excitons can reveal the correlated insulating states at fractional fillings^25–27^ and the intercell moiré exciton complexes^27^. The inter-moiré superlattice exciton interactions, however, remain an intriguing topic to pursue.

Experiment evidence has shown that the interlayer coherent carrier tunneling between electronic bands with the same spin in TMDC moiré superlattices can result in interlayer hybridized excitons, i.e., excitons with one of its constituent carriers delocalized across different layers in 2D heterostructures. This effect has been reported in MoSe_2_/WS_2_ and WSe_2_/WS_2_ moiré heterobilayers, as well as tunnel-coupled MoSe_2_ homo-bilayers separated by an h-BN spacer^5,18^, as long as the coherent tunneling is allowed and relevant exciton energies are tuned into resonance. Recently, a quadrupolar exciton state (Fig. 2e), which is composed of an electron in the middle layer and a hole delocalized between the top layer and the bottom layer^28^ was reported in angle-aligned TMDC heterotrilayers^29–33^. PL spectra suggest that the quadrupolar exciton exhibits an energy shift as a quadratic function of the out-of-plane electric field, in stark contrast to the linear energy shift expected from dipolar interlayer excitons in heterobilayers. The quadrupolar exciton can be explained by a two-level hybridization model considering the hybridization of two interlayer excitons with opposite dipole orientations, with a coupling strength around 9 − 30 meV and can be thus viewed as a superposition of the two dipolar excitons. A similar hybridization mechanism was also reported in trilayer WSe_2_/ monolayer WS_2_ moiré superlattices (Fig. 2f), where the hole tunneling between the first and the third WSe_2_ layer^34–36^ mixes the wavefunctions of two interlayer excitons from different moiré sites and one intralayer exciton, forming an exciton superposition across moiré sites^35^ that can be potentially used as a new type of qubit for quantum information processing.

The spin, valley, layer, and moiré site degrees of freedom of the TMDC moiré heterojunction will enable an even richer variety of excitonic complexes with versatile controls. Considering the existence of moiré flat bands, we expect the excitonic complexes in TMDC moiré heterojunctions to set up a new playground for strongly correlated physics, with many exciting results to be reported in the upcoming years.
